# Long-term medical utilization following ventilator-associated pneumonia in acute stroke and traumatic brain injury patients: a case-control study

**DOI:** 10.1186/1472-6963-11-289

**Published:** 2011-10-31

**Authors:** Chih-Chieh Yang, Nai-Ching Shih, Wen-Chiung Chang, San-Kuei Huang, Ching-Wen Chien

**Affiliations:** 1 Department of Emergency Medicine and Traumatology, Lotung Poh-Ai Hospital, No.83, Nan Chang St., Lotung, Yilan 265, Taiwan; 2 Institute of Health Policy and Management, National Taiwan University, No.17, Hsu-Chow Rd., Taipei, Taiwan; 3 Bureau of National Health Insurance, Department of Health, Executive Yuan, Taiwan, ROC, No.140, Sec. 3, Hsinyi Rd., Taipei, Taiwan; 4 Institute of Hospital and Health Care Administration, National Yang-Ming University, No.155, Sec. 2, Linong St., Beitou District, Taipei, Taiwan

**Keywords:** ventilator-associated pneumonia, medical utilization, longitudinal study, ICU, traumatic brain injury, acute hemorrhagic stroke, acute ischemic stroke

## Abstract

**Background:**

The economic burden of ventilator-associated pneumonia (VAP) during the index hospitalization has been confirmed in previous studies. However, the long-term economic impact is still unclear. The aim of this study is to examine the effect of VAP on medical utilization in the long term.

**Methods:**

This is a retrospective case-control study. Study subjects were patients experiencing their first traumatic brain injury, acute hemorrhagic stroke, or acute ischemic stroke during 2004. All subjects underwent endotracheal intubation in the emergency room (ER) on the day of admission or the day before admission, were transferred to the intensive care unit (ICU) and were mechanically ventilated for 48 hours or more. A total of 943 patients who developed VAP were included as the case group, and each was matched with two control patients without VAP by age ( ± 2 years), gender, diagnosis, date of admission ( ± 1 month) and hospital size, resulting in a total of 2,802 patients in the study. Using robust regression and Poisson regression models we examined the effect of VAP on medical utilization including hospitalization expenses, outpatient expenses, total medical expenses, number of ER visits, number of readmissions, number of hospitalization days and number of ICU days, during the index hospitalization and during the following 2-year period.

**Results:**

Patients in the VAP group had higher hospitalization expenses, longer length of stay in hospital and in ICU, and a greater number of readmissions than the control group patients.

**Conclusions:**

VAP has a significant impact on medical expenses and utilization, both during the index hospitalization during which VAP developed and in the longer term.

## Background

Nosocomial infections have a great impact on morbidity and mortality among hospitalized patients, and can significantly prolong hospital stay and increase medical expenses [1,2]. Among all nosocomial infections, hospital-acquired pneumonia is the second most frequent, being only marginally less common than urinary tract infection (UTI). Hospital-acquired pneumonia has the highest mortality rate and accounts for 60% of deaths associated with nosocomial infections [3]. Patients who are admitted to the intensive care unit (ICU) and receive invasive mechanical ventilation have the highest risk of nosocomial pneumonia, or ventilator-associated pneumonia (VAP). It has previously been reported that 10% to 24% of hospitalized patients who received mechanical ventilation for more than 48 hours will develop VAP [4,5]. In some developed countries the incidence of VAP has been reported to range from 6 to 37 per 1,000 ventilator-days [3,5-8], with a lower rate of 1.7 per 1,000 ventilator-days in long-term acute care hospitals [9]. The rates are higher in developing countries, ranging from 10 to 41.7 per 1,000 ventilator-days [10].

Many previous studies have found that VAP results in huge clinical and economic losses. The mortality rate of VAP patients is 30% to 50%, which is 2 to 8 times higher than the mortality rate without VAP [1,4,8,11-16]. Numerous studies have provided evidence that VAP can lead to significant increases in several important medical utilization items. When compared to patients without VAP, those who developed VAP spent an extra 6 to 22 days in ICU [3-5,8,12,15-19], were hospitalized for an extra 10 to 25 days [5,12,17], underwent an extra 5 to 12 days of ventilation [5,18,19], and cost an extra USD10,019 to USD41,000 in hospitalization expenses [4,5,12,17]. One Canadian study showed that an extra 17,000 ICU days per year could be attributed to VAP, which accounted for 2% of total ICU days and USD39.3 million in increased medical expenditure [3].

The incidence of VAP among hospitalized patients, especially critically ill patients, is substantial and causes significant negative outcomes. Previous studies on medical utilization in VAP have been limited to assessing medical expenses and the length of stay during the index hospitalization. The impact of VAP on long-term medical utilization is, however, still unclear. The main purpose of this study is to estimate the effects of VAP on medical expenses and utilization in the long term, after discharge from the index hospitalization. Previous studies did not focus specifically on patients who had the least possibility of being admitted with community-acquired pneumonia [8,12,15,16]. To avoid the confounding effects of community-acquired pneumonia or other hospital-acquired pneumonia, we selected only patients who were admitted to hospital with traumatic brain injury, acute hemorrhagic stroke, or acute ischemic stroke. A previous study indicated that trauma was a risk factor for the development of VAP[17], and that trauma patients are usually intubated emergently and then transferred to the ICU for further observation. This study therefore selected only acute stroke and traumatic brain injury patients who were transferred directly from the emergency room (ER) to the ICU, without admission to a ward. This should avoid the inclusion of cases of HAP which were acquired on the ward, and should reduce the possibility of over-estimation or under-estimation of medical utilization resulting from VAP.

## Methods

### Data source

This study analyzed data from the 2003-2006 National Health Insurance Research Database (NHIRD) of Taiwan, released by the National Health Research Institutes (NHRI) of Taiwan for public research purposes. The NHIRD is a database of all medical care claims data, including outpatient, ER and inpatient care, of more than 98% of the 23 million people of Taiwan. It is therefore one of the largest and most complete nationwide population-based datasets in the world. Information in this database includes diagnoses according to the International Classification of Diseases, 9th Revision, Clinical Modification (ICD-9-CM) codes, age, gender, detailed medical expenditures, treatments, prescriptions from each medical care interaction and hospital size. Data in the NHIRD that could be used to identify patients or care providers, including healthcare institutions and physicians, is encrypted before being sent to the NHRI for database construction and is encrypted again before being released to each researcher. Theoretically, it is impossible to query the data in a way that would identify individuals. All researchers who wish to use the NHIRD and its data subsets are required to sign a written agreement declaring that they do not intend to attempt to obtain information that could potentially violate the privacy of patients or care providers. This study was evaluated and approved by the NHRI for analysis (Application and Agreement Number: 99066 and 99167). The NHRI guidance committee granted ethical approval.

### Study subjects

The subjects included in the VAP (case) group were selected using the following steps. First we selected patients presenting with their first traumatic brain injury (ICD-9-CM codes 852, 853), acute hemorrhagic stroke (ICD-9-CM codes 430-432), or acute ischemic stroke (ICD-9-CM codes 433-436) who also had ICD-9-CM codes 481, 482, 483 and 486 (to identify those with pneumonia) during their index hospitalization. From this group, we selected those who were transferred directly from the ER to the ICU and received mechanical ventilation for 48 hours or more. Of these, we selected those who underwent endotracheal intubation in the ER on the day of admission or the day before admission. We used the definition of hospital-acquired pneumonia in the "Clinical Practice Guidelines for Pneumonia" published by NHRI, Taiwan [20]. This definition requires newly-developed infiltration on chest X-ray within 48 hours after admission, plus at least two of the following three conditions: 1) temperature increase of ≥1°C, or temperature ≥38.3°C or <35°C; 2) white blood cell count increase of >25%, or count of >10,000/μL or <3000/μL; or 3) >25 neutrophils per low-power field (×100) on Gram stain of sputum. The control group was selected from subjects without VAP. Each VAP case was matched with two control patients for age ( ± 2 years), gender, diagnosis at admission, date of admission ( ± 1 month) and hospital size. Both case and control groups excluded patients who died during the index hospitalization and who had a record of pneumonia during the year before admission, to facilitate the evaluation of medical utilization after discharge and to ensure that the pneumonia had developed after admission. A total of 1,367 patients met inclusion criteria for the case group and 4,241 met inclusion criteria for the control group. After matching, there were 943 eligible patients in the case group and 1,868 patients in the control group. These patients were followed up for 2 years after the index hospitalization. All of the 2,802 study subjects received positive pressure ventilation.

### Variables

The dependent variables of this study were medical expenses and utilization during the index hospitalization and during the 2-year period after discharge. These included hospitalization expenses, outpatient expenses, total medical expenses (sum of the expenses in outpatient departments, inpatient departments and ER), number of ER visits, number of readmissions, number of hospitalization days and number of ICU days. Medical expenses are presented in USD (average 2006 exchange rate 1.00 USD = 32.71 NTD). Among these dependent variables, outpatient expenses, total medical expenses, number of ER visits, and number of readmissions were only measured for the 2-year follow-up period. The main independent variable was whether or not a patient acquired VAP during the index hospitalization. Control variables were age, gender, diagnosis at admission, number of ventilation days during index hospitalization, basic health status of the patient, and hospital size of the index hospitalization. The basic health status was evaluated by the number of Elixhauser comorbidities [21] and whether or not there was a history of chronic respiratory disease. The hospital size was categorized as medical center (≥ 500 acute beds), regional hospital (500-250 acute beds) or district hospital (≤ 250 acute beds).

### Statistical analysis

SAS 9.1 software (SAS Institute Inc., Cary, NC) was used for all data processing and statistical analyses. Descriptive statistics including frequency, percentage, mean and standard deviation of the independent and dependent variables were calculated. Chi-square tests and t-tests were used to examine the differences between the two groups for the control and dependent variables. The impact of VAP on medical expenses and utilization was analyzed by regression models after adjusting for age, gender, diagnosis, ventilation days, hospital size, number of Elixhauser comorbidities and history of chronic respiratory disease. Since the medical expenses in our study violated the assumption of a normal distribution, we used robust regression to diminish the impact of outliers during the modeling of the effect of VAP on medical expenses, and also undertook logarithmic transformation. As the medical utilization measures were discrete and countable variables, Poisson regression models were applied to examine the effects of VAP on these variables. We described the results of Poisson regressions by exp (*β*). A *p*-value of less than or equal to 0.05 was considered to be statistically significant.

## Results

Table 1 shows patient characteristics and medical expenses and utilization data for all 2,802 study subjects. The diagnosis at admission was traumatic brain injury in 22.4%, acute hemorrhagic stroke in 43.4% and acute ischemic stroke in 34.2%. Among all study subjects, 67.5% were male, with a mean ( ± standard deviation, SD) age of 65.8 ± 15.1 years and 1.8 ± 1.7 Elixhauser comorbidities. The case group had a significantly longer duration of ventilation than the control group (16.1 vs. 12.7 days, *p *< 0.001). There was no significant difference in the prevalence of history of chronic respiratory disease between the two groups.

**Table 1:** Characteristics and medical utilization in the case and control groups

	Total (n = 2,802)	Case group (n = 943)	Control group (n = 1,868)	*p*-value
**Basic characteristics**				
Age ^a^, mean ± SD	65.8 ± 15.1	65.8 ± 15.1	65.8 ± 15.04	N.A.
Gender ^b^, n (%)				N.A.
Female	912(32.5)	304(32.5)	608(32.5)	
Male	1,890(67.5)	630(67. 5)	1,260(67. 5)	
Diagnosis at hospitalization ^b^, n (%)				N.A.
Traumatic brain injury	627(22.4)	209(22.4)	418(22.4)	
Acute hemorrhagic stroke	1,215(43.4)	405(43.4)	810(43.4)	
Acute ischemic stroke	960(34.2)	320(34.2)	640(34.2)	
Hospital size ^b^, n (%)				N.A.
Medical center	1,284(45.8)	428(45.8)	856(45.8)	
Regional hospital	1,302(46.5)	434(46.5)	868(46.5)	
District hospital	216(7.7)	72(7.7)	144(7.7)	
Chronic respiratory disease history ^b^, n (%)				0.751
No	2,159(77.1)	723(77.4)	1,436(76.9)	
Yes	643(22.9)	211(22.6)	432(23.1)	
Ventilation days ^a^, mean ± SD	13.8 ± 10.2	16.1 ± 10.5	12.7 ± 9.9	<0.001
Number of Elixhauser comorbidities ^a^, mean ± SD	1.8 ± 1.7	1.8 ± 1.7	1.8 ± 1.7	0.841
**Utilizations during the index hospitalization**				
Hospitalization expenses ^a^, mean ± SD	12,883 ± 9,016	14,063 ± 8,999	12,294 ± 8,969	<0.001
Hospitalization days ^a^, mean ± SD	42.4 ± 32.7	45.6 ± 33.2	40.8 ± 32.34	<0.001
ICU days ^a^, mean ± SD	17.7 ± 12.3	20.8 ± 12.9	16.2 ± 11.6	<0.001
**Utilizations during the follow-up period**				
Hospitalization expenses ^a^, mean ± SD	16,050 ± 23,000	17,840 ± 23,354	15,156 ± 22,774	0.004
Outpatient expenses ^a^, mean ± SD	3,274 ± 4,663	3,120 ± 4,863	3,350 ± 4,559	0.218
Total medical expenses ^a^, mean ± SD	19,324 ± 22,694	20,960 ± 23,114	18,507 ± 22,442	0.007
Number of ER visits ^a^, mean ± SD	1.7 ± 2.6	1.7 ± 2.6	1.7 ± 2.6	0.509
Number of readmissions ^a^, mean ± SD	4.2 ± 4.5	4.6 ± 4.6	4.0 ± 4.5	0.003
Hospitalization days ^a^, mean ± SD	111.1 ± 170.7	123.6 ± 174.3	104.8 ± 168.6	0.006
ICU days ^a^, mean ± SD	3.9 ± 11.0	4.7 ± 11.9	3.6 ± 10.5	0.007

^a ^t-test; ^b ^chi-square test

N.A. Not applicable

With regard to medical expenses and utilization, the patients with VAP had higher hospitalization expenses during both the index hospitalization (USD14,063 vs. USD12,294, *p *< 0.001) and the follow-up period (USD17,840 vs. USD15,156, *p *< 0.01), higher total medical expenses (USD20,960 vs. USD18,507, *p *< 0.01) and a greater number of readmissions (4.6 vs. 4.0, *p *< 0.01) during the follow-up period, more hospitalization days during both the index hospitalization (45.6 vs. 40.8, *p *< 0.001) and the follow-up period (123.6 vs. 104.8, *p *< 0.01), and more ICU days during both the index hospitalization (20.8 vs. 16.2, *p *< 0.001) and the follow-up period (4.7 vs. 3.6, *p *< 0.01).

The results of the regression models estimating the impact of VAP on medical utilization during the index hospitalization with adjustment for demographic variables, basic health status, and characteristics of the index hospitalization are shown in Table 2. Hospitalization expenses were 5.9% higher in the VAP group than in the control group. Hospitalization days were increased by 0.3% and ICU days were increased by 13.2% in the VAP group. Diagnosis, hospital size, history of chronic respiratory disease, ventilation days and number of Elixhauser comorbidities were also significantly associated with medical utilization during the index hospitalization.

**Table 2:** Regression models of medical utilization during the index hospitalization

	Hospitalization expenses ^b^	Hospitalization days ^c^	ICU days ^c^
	β	β	β
VAP			
No (control group) ^a^			
Yes (case group)	1.059**	0.003***	0.132***
Age	0.999	-0.001***	0.001*
Gender			
Female ^a^			
Male	1.010	0.018**	-0.005
Diagnosis at hospitalization			
Traumatic brain injury ^a^			
Acute hemorrhagic stroke	1.087***	0.056***	0.075***
Acute ischemic stroke	0.970	0.117***	0.046**
Hospital size			
Medical center ^a^			
Regional hospital	0.746***	-0.157***	-0.036***
District hospital	0.476***	-0.387***	-0.228***
Chronic respiratory disease history			
No ^a^			
Yes	0.922**	-0.045***	-0.027*
Ventilation days	1.036***	0.023***	0.032***
Number of Elixhauser comorbidities	1.022***	0.028***	0.010**
R-Square	0.353		

^a ^Reference group; ^b ^robust regression; ^c ^Poisson regression

*p < 0.05; **p < 0.01; ***p < 0.001

Table 3 shows that number of readmissions was 5.7% higher in the VAP group than in the control group. Hospitalization days were increased by 5.4% and ICU days were increased by 26.5% in the VAP group. However, no significant differences were observed in hospitalization expenses, outpatient expenses, total medical expenses or the number of ER visits. Patients who were older, male, had a diagnosis of stroke, had a greater number of ventilation days, and had more Elixhauser comorbidities were found to have a higher rate of medical utilization.

**Table 3:** Regression models of medical utilization during the 2-year follow-up period

	Hospitalization expenses ^b^	Outpatient expenses ^b^	Total medical expenses ^b^	Number of ER visits ^c^	Number of readmissions ^c^	Hospitalization days ^c^	ICU days ^c^
	β	β	β	β	β	β	β
VAP							
No (control group)^a^							
Yes (case group)	1.103	0.934	1.064	-0.013	0.057**	0.054***	0.265***
Age	1.006**	0.995***	1.011***	0.005***	0.008***	0.008***	0.020***
Gender							
Female ^a^							
Male	1.229**	0.978	1.257***	0.256***	0.178***	0.134***	0.420***
Diagnosis at hospitalization							
Traumatic brain injury ^a^							
Acute hemorrhagic stroke	1.264**	1.543***	1.465***	0.124**	0.217***	0.333***	0.462***
Acute ischemic stroke	1.411***	1.088	1.438***	0.083	0.203***	0.373***	0.622***
Hospital size							
Medical center ^a^							
Regional hospital	0.915	0.885	0.946	-0.034	-0.032	-0.124***	0.211***
District hospital	0.924	0.615***	0.792*	-0.298***	-0.056	-0.131***	0.296***
Chronic respiratory disease history							
No ^a^							
Yes	0.951	0.810**	0.856*	-0.068	-0.040	-0.022***	-0.030
Ventilation days	1.034***	0.990***	1.031***	-0.009***	0.019***	0.030***	0.007***
Number of Elixhauser comorbidities	1.072***	1.040*	1.086***	0.066***	0.031***	0.005***	0.105***
R-Square	0.091	0.028	0.087				

^a^Reference group; ^b ^robust regression; ^c ^Poisson regression

*p < 0.05; **p < 0.01; ***p < 0.001

## Discussion

This study expands on the existing evidence that VAP increases medical expenses and utilization during the index hospitalization by exploring the impact of VAP for a 2-year period following discharge. Analysis of longitudinal data shows that VAP results in substantially increased medical expenses and utilization in the longer term. The use of a nation-wide database, namely the NHIRD, which includes information regarding diseases, medical expenditures and utilization for more than 98% of the population of Taiwan, enabled us to include cross-hospital analysis.

Even though the differences between the VAP and control groups were not large enough to be clinically significant in some of the dependent variables with statistical significance, such as the number of readmissions, we still believe that the impact of VAP is considerable from a population perspective. We found that outpatient expenses were not higher in the VAP group than in the control group. To further explore this paradoxical phenomenon, we further evaluated the data and found that some VAP patients had been discharged to nursing homes of the same hospital since the hospitalization. These VAP patients therefore did not utilize outpatient services, resulting in lower outpatient expenses in this group.

In both the case and control groups, medical expenses were lower than figures given by prior studies undertaken in other countries. This is expected because the cost of medical care is lower in Taiwan than in most Western countries [17]. For example, the average cost per ICU day in Canada was estimated at USD1,978 (NTD 64,692) [3], while the highest cost per ICU day in Taiwan in our study was USD196 (NTD6,400).

Previous studies have indicated that the impact of VAP is not limited to the duration of hospitalization, but will affect many aspects of patients' lives after discharge as well as society as a whole. Areas affected include increased antibiotic costs, increased microbial resistance, the development of secondary infections, and the development of various other complications [3]. In addition to a longer length of stay and higher medical expenditure, patients who develop a nosocomial infection have been found to have a higher risk of complications including shock, renal failure, respiratory failure, irreversible ventilator support, heart failure, neurological failure, liver failure and death [1]. Our results are consistent with these previous findings, showing that VAP has a long term impact on medical expenses and utilization, which may be a consequence of these complications. Further studies are needed to investigate the long-term pathophysiological effects of VAP.

There is ongoing discussion exploring whether increased medical utilization is a consequence of VAP, or simply a feature of the frailer patients who have a higher risk of acquiring VAP. This can be evaluated using well-matched VAP and control groups. We designed a case-control study with strict matching of variables including age, gender, diagnosis, date of admission and hospital size, and were therefore able to eliminate the confounding effects of these characteristics on medical expenses and utilization, and enhance comparability between the case and control groups. We found no significant differences in the prevalence of history of chronic respiratory disease or in the number of Elixhauser comorbidities between the case and control groups, confirming that the groups were well matched.

Some limitations of this study should be noted. First, the accuracy of the VAP diagnosis could not be verified by directly examining medical records or laboratory reports, due to the limitation of the NHIRD being an administrative database. We were not able to obtain specific VAP information from the ICD-9-CM codes of the NHIRD. However, the definition of VAP in this study was very strict; subjects in the case group all underwent endotracheal intubation in the ER on the day of admission or the day before admission, were admitted directly from the ER to the ICU, received mechanical ventilations for 48 hours or longer, and developed pneumonia. As the patients were admitted directly from the ER to the ICU without admission to a ward, we were able to exclude other causes of hospital-acquired pneumonia. We have confidence in the diagnoses because the Bureau of National Health Insurance (BNHI) has established strict review procedures to avoid miscoding and inappropriate claims. Second, we lack information on disease severity and other basic physical characteristics, such as Glasgow Coma Score, stroke scale, swallowing function and adequacy of cough, which may have an impact on the level of medical utilization. To correct this potential bias, we included three variables in the regression models as proxies for underlying health condition: ventilation days, the number of Elixhauser comorbidities, and a history of chronic respiratory disease. In the multivariable analysis models, we adjusted these confounders to investigate the individual effect of VAP on medical expenses and utilization. This is an advantage of the present study since most previous studies evaluated the economic outcomes of VAP using case-control studies with no adjustment for potential confounders [1,5,18,19].

The impact of VAP on medical expenses not only causes considerable economic burdens on health insurers, but also results in substantial losses for hospitals. Even though the Unites States and Germany have different healthcare systems, studies in both countries have demonstrated that the medical expenses reimbursed by health insurers for nosocomial infections were far less than the total cost to the hospitals [19,22]. The importance of VAP prevention is therefore not limited to improving the health outcomes of patients and their quality of care. For hospitals, the prevention of VAP has great significance in terms of cost containment and efficient medical care delivery. According to one study in Canada, a 50% reduction in the incidence of VAP could reduce medical expenditure by about USD21.4 million per year [3]. As the data analyzed in the present study were National Health Insurance claims data, we were only able to calculate the expenses reimbursed by the BNHI of Taiwan, and other expenditures covered by hospitals and patients were not included. The total impact of VAP on medical expenses is therefore likely to be even higher than the estimation presented in this study.

## Conclusions

In conclusion, VAP has a significant impact on medical expenses and utilization, during both the index hospitalization and in the longer term. As in other developed countries, Taiwan is confronted with the pressures of an aging population and advances in medical technology for the treatment of critically ill patients. In this context, the use of mechanical ventilation is likely to increase in the future, which further increases the importance of VAP prevention. Hospital administrators and health policy makers have a responsibility to enhance their efforts to prevent VAP, both to improve quality and outcomes of medical care and to contain costs.

The major finding of this study is that the significant impact of VAP on medical expenses and utilization continues beyond the index hospitalization to the longer term.

## List of abbreviations

VAP: ventilator-associated pneumonia; ICU: intensive care unit; ER: emergency room; UTI: urinary tract infection; NHIRD: National Health Insurance Research Database; NHRI: National Health Research Institutes; ICD-9-CM: International Classification of Diseases, 9th Revision, Clinical Modification; SD: standard deviation; BNHI: Bureau of National Health Insurance.

## Competing interests

The authors declare that they have no competing interests.

## Authors' contributions

CCY conceived of the study concept and analyzed the data. NCS assisted with data analysis and interpretation of results. WCC drafted and revised the manuscript. SKH managed the data and helped to revise the manuscript. CWC designed the study, and supervised the data analysis and interpretation of results. All authors read and approved the final manuscript.

## Pre-publication history

The pre-publication history for this paper can be accessed here:

http://www.biomedcentral.com/1472-6963/11/289/prepub
